# Comparison of the ALPS and PHILOS plating systems in proximal humeral fracture fixation – a retrospective study

**DOI:** 10.1186/s12891-023-06477-9

**Published:** 2023-05-10

**Authors:** Antoine Dewarrat, Alexandre Terrier, Bardia Barimani, Frédéric Vauclair

**Affiliations:** 1 University of Lausanne, Lausanne, Switzerland; 2 Department of Orthopaedics and Traumatology, Lausanne University Hospital, Lausanne, Switzerland; 3 Laboratory of Biomechanical Orthopedics, Ecole Polytechnique Fédérale de Lausanne (EPFL), Station 9, 1015, Lausanne, Switzerland; 4 Division of Orthopedic Surgery, McGill University, Montreal, QC Canada; 5 University of Toronto, Toronto, Canada

**Keywords:** Proximal humerus fracture, Plate, ALPS, PHILOS, Fracture fixation, Locking plate

## Abstract

**Background:**

Open reduction and plate osteosynthesis are considered as a successful technique for the treatment of proximal humerus fracture (PHF) despite high complication rates. The objective of our study was to review the clinical outcome and complications of the Anatomic Locking Plate System (ALPS) and compare it to the Proximal Humeral Internal Locking System (PHILOS). Our hypothesis was that ranges of motion (ROM) were superior and complication rates were lower with ALPS.

**Methods:**

Twenty patients treated with ALPS for PHF were retrospectively compared to 27 patients treated with PHILOS. Union, ROM and complications were clinically and radiologically assessed at 6 weeks, 3, 6, 12 and 18–24 months post-operatively.

**Results:**

Mean age was 52 ± 14 in the ALPS group and 58 ± 13 in the PHILOS group. Last follow-ups were conducted at a mean of 20.6 ± 4.8 months. Mean shoulder abduction was superior with ALPS by 14° (*p*-value = 0.036), 15° (*p*-value = 0.049), and 15° (*p*-value = 0.049) at 3, 6, and 12 months respectively. Mean shoulder external rotation was superior with ALPS by 11° (*p*-value = 0.032), 15° (*p*-value = 0.010) and 12° (*p*-value = 0.016) at 6 weeks, 3 and 6 months respectively. At the end of the follow-up, ROM remained better with ALPS, but not significantly. Complication rates over 21 months reached 20% with ALPS and 48% with PHILOS (*p*-value = 0.045). Implant removal rates reached 10% with ALPS and 37% with PHILOS (*p*-value = 0.036). Avascular necrosis was the only cause for hardware removal in the ALPS group.

**Conclusion:**

The ALPS group showed better clinical outcomes with faster recovery in abduction and external rotation, although no difference in ROM remained after 21 months. Additionally, the complications rate was lower at last follow up. In our experience, the ALPS plating system is an effective management option in some PHF.

## Introduction

Proximal humerus fracture (PHF) is the third most common fracture and accounts for approximately 5% of all adult fractures [1, 2]. PHF commonly affects elderly females due to osteoporosis [3], with a rising incidence after the age of 50 years, and peak after 80 years [1]. With an aging population, a continuous increase in this type of fracture can be expected in the upcoming years and rates estimated to triple between 2008 and 2030 [4]. Along with the expected increase in PHF, understanding the optimal management of this pathology becomes ever more important.

In 47% to 80% of PHF cases, the fracture is non or minimally displaced [1, 5] and is amenable to conservative management with satisfying results [6]. However, fractures including complex morphologies, dislocation or significant displacement may benefit from surgical intervention in selected patients [7]. This remains controversial as some studies question the superiority of operative versus non-operative management of PHF [8–10].

Despite the absence of consensus, surgical management remains common practice and various techniques exist, such as nailing, cabling, arthroplasty, and the use of locking plates [11]. Elderly patients with osteoporotic bone mostly benefit from arthroplasty [7, 12], whereas younger patients are mainly treated through closed reduction and internal fixation with intramedullary nailing or open reduction and plate osteosynthesis to achieve satisfactory function [13–17].

Intramedullary nail fixation provides superior performance in terms of stiffness and load to failure in comparison to locking plates [18, 19]. However, rotator cuff and cartilage injuries are risks during nail insertion and shoulder function may be compromised [20]. Hardware-related complications are relatively common and range from 9.3% to 70% [20, 21], with hardware removal required in 7% to 15% of cases [20, 22]. Some studies suggest similar performance and complication rate with nail and locking plates [23, 24] while others suggest superiority with nails [25] and thus further research is required on this subject. Additionally, with the lack of consensus regarding adverse event terminology among various operative shoulder options, it becomes difficult to compare them together [26].

Despite the lack of unanimity on optimal treatment of PHF, locking plates are one of the most advanced options [27]. It is considered by many as a successful technique bringing more stability and improving shoulder ranges of motion (ROM) [7, 24, 28]. However, one of the main drawbacks with locking plates is the high complication rate [27, 29], and studies have reported complication rates of 33% in 7,182 patients [30] and up to 49% in 514 patients [31]. Almost half of the complications are implant-related, with the majority being attributed to screws [30]. According to Sproul et al. [31], varus malunion, subacromial impingement and screw perforation represent 30% of the complications and reoperation rate with locking plates reaches as high as 14% [31, 32].

**Fig. 1:**
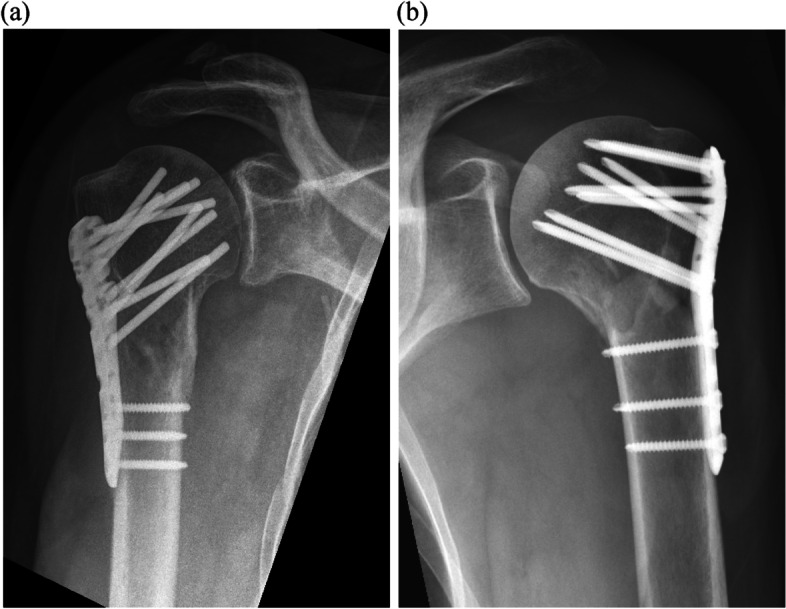
Plate sitting lower to the greater tuberosity, smooth blunt-ended pegs and calcar screws positioned in the inferior cortex with the ALPS plating system (**a**) may allow to reduce complications in comparison to plate sitting higher and fixed trajectory calcar screw (**b**)

The Anatomic Locking Plate System (ALPS) (Biomet) (Fig. 1) offers multiple features aiming to address the main complications of locking plates, such as variable angle calcar screws, low-profile plates, and smooth blunt-ended pegs [33]. Variable angle calcar screws provide additional fixation in the inferior medial cortex to avoid varus collapse and malunion. Multi-directional locking screws allow for up to 25° cone of angulation and better screw positioning. Low-profile plates sit at 20 mm distal to the top of the greater tuberosity to avoid subacromial impingement. Smooth blunt-ended pegs replace screws (optional) in the humeral head to minimize articular surface screw perforation.

**Fig. 2:**
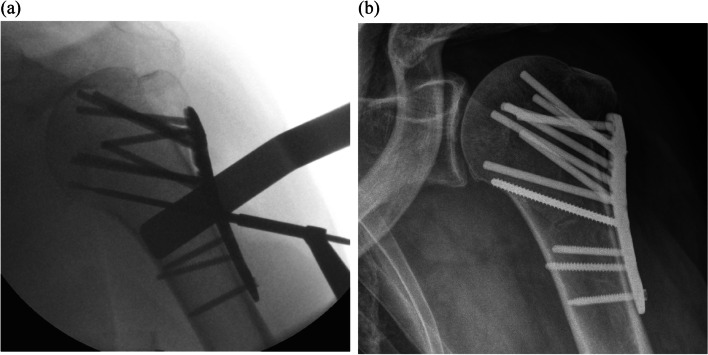
Intra-operative (**a**) and post-operative (**b**) radiographs of variable angle calcar screw positioning in the inferior cortex with the ALPS plating system

These features are available with the ALPS plating system, but not with the Proximal Humeral Internal Locking System (PHILOS) (Depuy-Synthes) (Fig. 1) which may explain the difference in complication rate. The advantages of medial calcar screws in providing stability and maintaining reduction has been widely demonstrated [34–36]. It has also been suggested that the lack of screws in the calcar due to a fixed trajectory locking screw has a negative impact on fixation strength [37]. While the PHILOS plating system only offers fixed trajectory locking screws [38], the multi-directional locking screws available with the ALPS plating system may allow better positioning of the screw inside the calcar (Fig. 2), providing improved construct stability and avoiding secondary displacement potentially leading to malunion. The low-profile plate of the ALPS plating system sits lower than the PHILOS plate at 20 mm distal to the top of the greater tuberosity [33] in comparison to 5–7 mm respectively [38], which may be the reason for lower subacromial impingement rates [39, 40]. Finally, the smooth blunt-ended pegs available with the ALPS plating system may lower articular surface screw perforation. The ALPS plating system has reported equitable union rates, time to union and functional scores compared to other plating system over 31.9 weeks [39] and lower complication rates compared to the PHILOS plating system [40]. However, these studies were limited in terms of follow-up period, number of patients or ethnic group.

Considering that the additional features of the ALPS plating system could lead to better clinical outcomes, the aim of the present retrospective study is to review the performance and safety of the ALPS plating system for the treatment of PHF after a follow-up period of at least 18 months and compare it to the PHILOS plating system. Following previous results of the ALPS plating system [39, 40], we decided to test the hypothesis that ROM were superior and complication rates lower with the ALPS plating system compared to the PHILOS plating system.

## Methods

### Population

A consecutive series of 20 patients operated between February 2017 and September 2018, treated with the ALPS plating system for PHF were retrospectively selected and reviewed. A consecutive series of 27 patients operated between March 2015 and December 2016, treated with the PHILOS plating system for the same indication were included for comparison. Inclusion criteria were a displaced fracture of the proximal humerus (type 11 according to the AO classification system [41]) and an age of 18 years old or older. Exclusion criteria was ipsilateral upper limb arterial injuries.

This study was approved by the Human Research Ethics Committee of the Canton Vaud CER-VD (Date 30.12.2020 / No 2020–01,292). Broad written informed consent was obtained from participants for research studies. If not available, study-specific verbal informed consent was obtained.

### Surgical intervention and post-op rehabilitation

All surgeries were performed directly or under supervision of the same fellowship trained upper extremity trauma surgeon (FV). All patients were positioned in the beach chair (except one polytrauma patient which was operated on in the supine position). A delto-pectoral approach was used in all cases. Following open reduction and temporary fixation with K-wires, the quality of reduction was checked under fluoroscopic imaging according to Schnetzke et al. [36]. The plate position was then assessed before drilling the screws and final results were evaluated using fluoroscopic guidance to check screw length and position. Lastly, fractured tuberosities were secured to the plate using number 2 Fiberwire (Arthrex). Patients were protected by a sling for 6 weeks and rehabilitation with a standardized protocol was started on post-operative day 1.

### Clinical evaluation

Post-operative clinical follow-ups were performed by an orthopaedic trauma surgeon and were scheduled at 6 weeks, followed by 3, 6, 12 and 18 or 24 months after surgery in our trauma center. Clinical union, ROM and complications were documented at each follow-up. ROM included shoulder flexion, abduction, external rotation and internal rotation. When ROM was reported as “full”, we converted “full” to a numerical value as follows:

- full *flexion* was set at 151°, the lowest value of the maximum amplitude range according to orthopaedic scores [42];
- full *abduction* was set at 151°, the lowest value of the maximum amplitude range according to orthopaedic scores [42];
- full *external rotation* was set and adjusted to age, sex and side according to Gill et al. [43];
- full *internal rotation* was set at T7 vertebrae, the maximum amplitude according to orthopaedic scores [42]. As internal rotation was constantly reported descriptively (e.g. T7 vertebrae), we converted internal rotation to a numerical scale from 1 to 20: 1–3 greater trochanter, gluteus maximus and sacrum respectively; 4–8 for distal to proximal lumbar vertebrae; 9–20 for distal to proximal thoracic vertebrae.

### Radiographic evaluation

Pre-operative X-rays were retrospectively collected, and fractures were classified according to the Neer [5] and AO [41] classification systems. Post-operative X-rays were subsequently obtained at each follow-up (AP and lateral views) and were retrospectively assessed for union and complications. All radiographic analyses were supervised by the same fellowship trained upper extremity trauma surgeon (FV).

### Complications

Intra-operative surgical complications were documented for comparison. Complication rates included both clinical and radiographic aspects and were documented as number of patients with at least one complication, number of complications across all patients and number of complications by type. All complication rates were defined over the follow-up period. Types of complication were recorded as secondary displacement, non-union, avascular necrosis, screw perforation, subacromial impingement, infection, plate failure, nerve palsy, pulmonary embolus and stiffness. Non-union was defined as absence of bone consolidation after 6 months on post-operative X-rays. In case of implant removal, ROM was documented at the last follow-up prior to implant removal and at the first follow-up after removal.

### Statistical analysis

We verified the matching of gender, age, BMI, affected side, follow-up length or fracture morphology, between the ALPS and PHILOS groups, with Wilcoxon and Fisher tests. Since some variables did not follow a normal distribution, and because of the relatively limited sample size, we used the non-parametric one-sided and two-sample Wilcoxon tests (Mann–Whitney) to test the hypothesis that the ROM (flexion, abduction, external rotation, and internal rotation) were superior for the ALPS than PHILOS plates. We reported effect size (ES) with 95% confidence interval, and *p*-value. To evaluate the difference of complication rates, and implant removal, between the ALPS and PHILOS plates, we used Fisher's exact test, and reported odd ratio (OR) with 95% confidence interval, and *p*-value. The normality of the data was evaluated with Shapiro–Wilk test. The statistical analyses were performed with R 4.0 (R Foundation for Statistical Computing, Vienna, Austria. www.R-project.org).

## Results

### Population

Twenty patients (mean age 52 ± 14) treated with ALPS (A) plates were compared to 27 patients (mean age 58 ± 13) treated with PHILOS (P) plates. Both groups were not significantly different in terms of patient number, gender, age, BMI, affected side, follow-up length or fracture morphology (Table 1). There were 13 smokers (A 6, P 7) and 9 alcohol consumers (A 5, P 4) excluding occasional or former consumers. No patient had previous shoulder surgery. Mechanism of injury showed no difference (*p*-value = 0.768) and was distributed in each group as follows: simple fall (A 50%, P 44%), sporting injury (A 20%, P 33%), public road accident (A 15%, P 11%), fall from less than 3 m (A 10%, P 11%), fall from more than 3 m (A 5%). Five (A 3, P 2) patients suffered from polytrauma. One patient had bilateral fractures. One had osteopenia.

**Table 1:** Patient characteristics according to treatment group

	ALPS	PHILOS	*p*-value
Number of patients (male / female)	20 (11 / 9)	27 (16 / 11)	1.000
Age at surgery (mean ± sd years)	52$\pm$14	58$\pm$13	0.140
BMI at surgery (mean ± sd kg/m^2^)	26$\pm$4	26$\pm$5	0.864
Right / left affected ratio (%)	9 / 11 (45 / 55)	13 / 14 (48 / 52)	1.000
Follow-up length (mean ± sd months)	22 ± 5	19 ± 4	0.129
Neer classification (%)			
2-part	6 (30)	12 (44)	0.374
3-part	10 (50)	9 (33)	0.368
4-part	4 (20)	6 (22)	1.000
AO classification^a^ (%)			
11.A2	2 (10)	0 (0)	0.176
11.A3	3 (15)	9 (33)	0.191
11.B1	3 (15)	3 (11)	1.000
11.B3	1 (5)	1 (4)	1.000
11.C1	6 (30)	1 (4)	0.032
11.C2	4 (15)	9 (33)	0.348
11.C3	1 (5)	3 (11)	0.626
Duration of surgery^b^ (mean ± sd min)	165$\pm$35	187$\pm$69	0.270
Additional ipsilateral injury			
other fracture	2	1	0.567
neurological	2^c^	0	1.000
vascular	0	0	1.000
pulmonary	2	1	0.567

^a^One pre-operative X-ray missing

^b^Two exclusions for additional surgical procedures other than on shoulder, two exclusions for bilateral procedure, one missing for unknown reason

^c^One hypoesthesia of the ulnar and radial nerves areas, one plexopathy

### Follow-ups

Out of 47 patients, 91% (A 18, P 25) were available for follow-up at 6 weeks, 94% (A 17, P 27) at 3 months, 94% (A 19, P 25) at 6 months, 72% (A 17, P 17) at 12 months and 60% (A 15, P 13) at 18–24 months. One patient was discharged before completing the 18–24 months’ follow-up due to full recovery and one by request of the patient due to satisfactory function. Retrospectively, post-operative clinical follow-ups were conducted at a mean of 6.0 ± 1.3 weeks, 2.9 ± 0.4 months, 6.0 ± 1.0 months, 11.9 ± 1.0 months and 20.6 ± 4.8 months.

### Ranges of motion

**Fig. 3:**
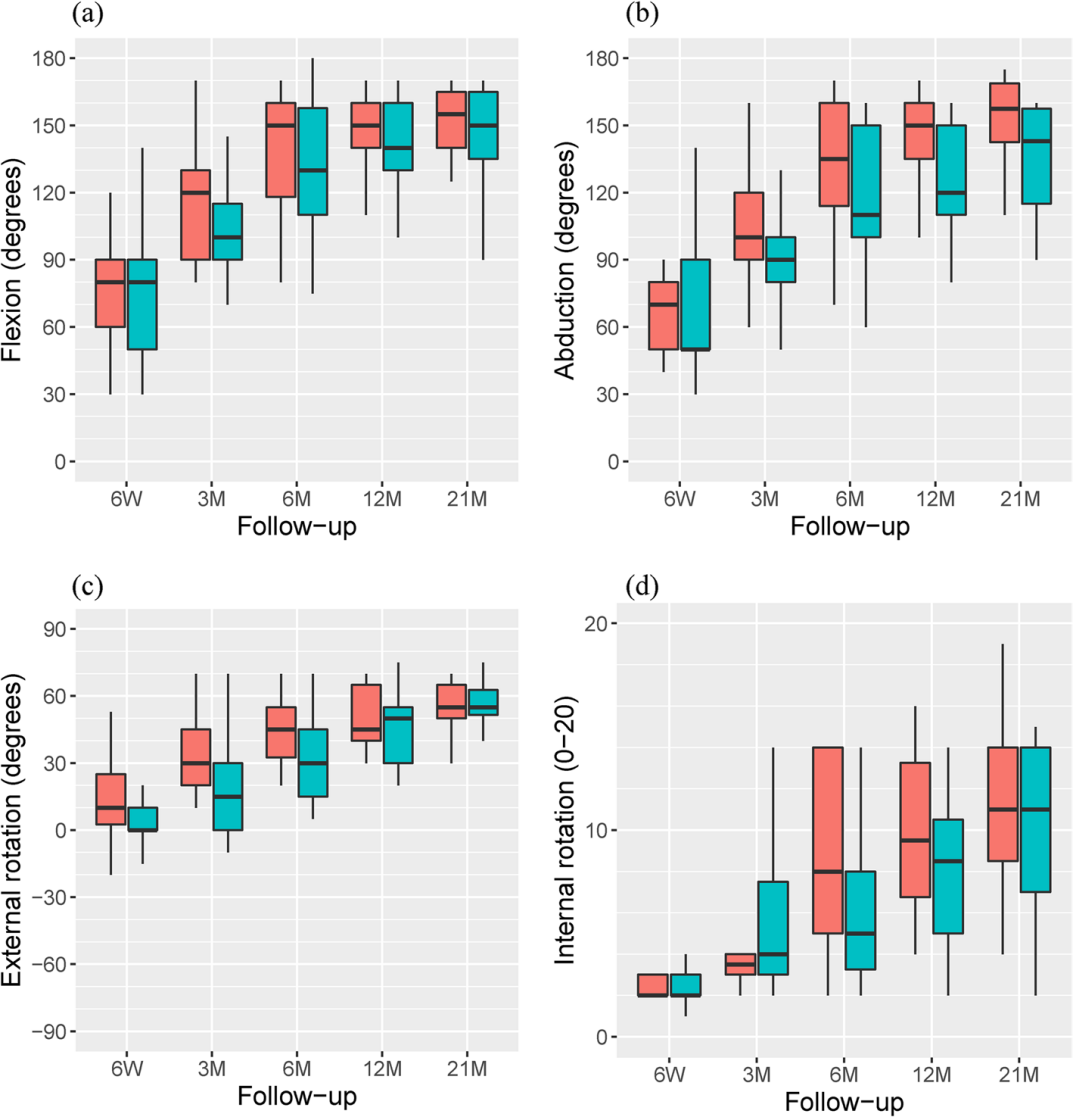
Shoulder flexion (**a**), abduction (**b**), external rotation (**c**) and internat rotation (**d**) in the ALPS (orange) and PHILOS (blue) group at 6 weeks, 3, 6, 12 and 21 months after surgery. Boxplots include minimum, maximum, median, first and third quartiles

While mean *flexion* was predominantly higher in the A group, there was very weak evidence with a small effect size between 3 and 21 months for the superiority of the A group in comparison to the P group (Table 2, Fig. 3).

**Table 2:** Range of motion

	Mean		Median (IQR^a^)		Effect size (95% CI)	*p*-value
	ALPS	PHILOS	ALPS	PHILOS		
Flexion (°)						
at 6 weeks	73$\pm$21	74$\pm$27	80 (30)	80 (40)	0.002 (0.01, 0.33)	0.510
at 3 months	115$\pm$25	101$\pm$30	120 (40)	100 (25)	0.21 (0.01, 0.49)	0.080
at 6 months	138$\pm$28	130$\pm$29	150 (40)	130 (41)	0.13 (0.01, 0.44)	0.140
at 12 months	143$\pm$27	134$\pm$35	150 (20)	140 (20)	0.14 (0.01, 0.48)	0.130
at 21 months	146$\pm$26	139$\pm$39	155 (25)	150 (30)	0.004 (0.001, 0.4)	0.322
Abduction (°)						
at 6 weeks	66$\pm$17	65$\pm$26	70 (30)	50 (40)	0.06 (0.01, 0.39)	0.358
at 3 months	103$\pm$26	89$\pm$25	100 (30)	90 (20)	0.27 (0.02, 0.54)	0.036
at 6 months	131$\pm$32	116$\pm$29	135 (40)	110 (50)	0.24 (0.02, 0.52)	0.049
at 12 months	137$\pm$33	122$\pm$33	150 (25)	120 (38)	0.27 (0.02, 0.62)	0.049
at 21 months	147$\pm$29	126$\pm$42	158 (26)	141 (41)	0.31 (0.04, 0.64)	0.058
External rotation (°)						
at 6 weeks	14$\pm$20	3$\pm$10	10 (23)	0 (10)	0.36 (0.03, 0.68)	0.032
at 3 months	33$\pm$16	18$\pm$21	30 (25)	15 (30)	0.36 (0.08, 0.60)	0.010
at 6 months	45$\pm$15	33$\pm$18	45 (23)	30 (30)	0.32 (0.07, 0.56)	0.016
at 12 months	49$\pm$13	45$\pm$17	45 (25)	50 (25)	0.06 (0.01, 0.42)	0.380
at 21 months	57$\pm$14	51$\pm$24	55 (15)	55 (11)	0.01 (0.01, 0.41)	0.539
Internal rotation (1–20)						
at 6 weeks	4$\pm$4	3$\pm$2	2 (1)	2 (1)	0.14 (0.01, 0.52)	0.270
at 3 months	5$\pm$4	5$\pm$3	4 (1)	4 (5)	0.13 (0.01, 0.47)	0.773
at 6 months	8$\pm$4	6$\pm$4	8 (9)	5 (5)	0.25 (0.01, 0.52)	0.060
at 12 months	10$\pm$4	8$\pm$4	10 (7)	9 (6)	0.20 (0.01, 0.52)	0.139
at 21 months	11$\pm$5	10$\pm$4	11 (6)	11 (7)	0.1 (0.01, 0.48)	0.312

^a^Interquartile range

Mean *abduction* was superior in the A group with a small effect size between 3 and 12 months. Mean *abduction* was higher by 14°, 15° and 15° at 3, 6 and 12 months respectively in comparison to the P group. There was very weak evidence as well for an increase of 21° in abduction with a moderate effect size at 21 months in comparison to the P group.

Mean *external rotation* was superior in the A group with a moderate effect size between 6 weeks and 6 months. Mean *external rotation* was higher by 11°, 15° and 12° at 6 weeks, 3 and 6 months respectively in comparison to the P group.

While mean *internal rotation* was predominantly higher in the A group, there was only very weak evidence with a small effect size between 6 weeks and 21 months for the superiority of the A group in comparison to the P group.

Despite a moderate effect size for abduction and better ranges of motion, no significant difference remained at 21 months.

### Complications

No intra-operative surgical complications were reported. Twenty-eight post-operative complications among 17 patients (A 4, P 13) were reported (Table 3). In the A group, 20% (4/20) of patients presented at least one complication over 21 months in comparison to 48% (13/27) in the P group (OR = 0.277 [0.000, 0.970], *p*-value = 0.045). Whilst not statistically different, screw perforation was the predominant complication in both groups (A 10%, P 22%) (Table 3). Other main complications in each group included secondary displacement (A 10%, P 15%) and avascular necrosis (A 10%, P 7%). The following complications were only seen in the P group: subacromial impingement (11%), nerve palsy (7%) (1 transitory hypoesthesia of the median nerve secondary to the supra-clavicular catheter, 1 hypoesthesia of the lateral cutaneous nerve of the forearm without motor deficit), stiffness (7%), infection (7%) and pulmonary embolus (4%) (Table 3). Plate failure and non-union were not reported.

**Table 3:** Complication rates over 21-month follow-up period

	ALPS	PHILOS	Odd ratio (95% CI)	*p-*value
Number of patients with at least one complication (% of patients)	4 (20)	13 (48)	0.277 [0.000 -0.970]	0.045
Number of complications by type (% of patients)				
secondary displacement	2 (10)	4 (15)	0.65 (0.00, 3.88)	0.489
avascular necrosis	2 (10)	2 (7)	1.38 (0.14, inf.)	0.574
screw perforation	2 (10)	6 (22)	0.40 (0.00, 2.04)	0.242
subacromial impingement	0 (0)	3 (11)	0.00 (0.00, 2,27)	0.180
infection	0 (0)	2 (7)	0.00 (0.00, 4.67)	0.325
non-union	0 (0)	0 (0)	-	-
plate failure	0 (0)	0 (0)	-	-
nerve palsy	0 (0)^a^	2 (7)	0.00 (0.00, 4.67)	0.325
pulmonary embolus	0 (0)	1 (4)	0.00 (0.00, 25.7)	0.575
stiffness	0 (0)	2 (7)	0.00 (0.00, 4.67)	0.325

^a^One suffered from pre-operative nerve palsy persisting after reduction

In the A group, 2/20 patients (10%) required implant removal during the follow-up period compared to 10/27 patients (37%) in the P group (OR = 0.195 [0.000, 0.901], *p*-value = 0.036) (Table 4). Avascular necrosis was the only cause for implant removal in the A group and counted for 20% of removal in the P group. Other causes in the P group included screw perforation (50%), impingement (20%) and stiffness (10%).

**Table 4:** Implant removal

	ALPS	PHILOS
Number of patients with implant removal (%)	2 (10)	10 (37)
Causes (% of patients)		
avascular necrosis	2 (10)	2 (7)
screw perforation	0 (0)	5 (19)
impingement	0 (0)	2 (7)
stiffness	0 (0)	1 (4)

## Discussion

Our analysis to review the performance and safety of the ALPS plating system was based on post-operative clinical and radiological data including 47 patients with a mean follow-up of 21 months. Our results suggest the ALPS plating system is superior to the PHILOS plating system in terms of abduction, external rotation and complication rates.

Regarding ROM, flexion, abduction, external rotation and internal rotation were higher in the ALPS group and for all the follow-up periods but the difference was statistically significant only for *abduction* and *external rotation.* After 21 months, no significant difference remained. In other words, the ALPS plate was associated with a statistically faster recovery, what may be explained by the ALPS plate sitting lower than the PHILOS plate. We did not see any statistical difference regarding functional outcome at last follow-up.

**Fig. 4:**
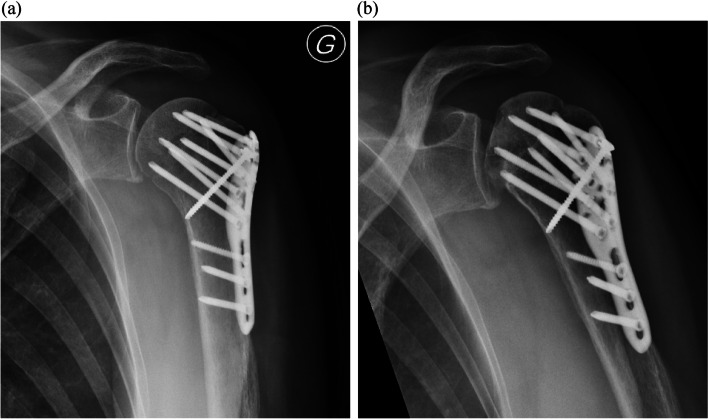
Long follow-up period allowed to report late complications with this patient with no complication at 6 months (**a**) and avascular necrosis at 12 months (**b**)

Complication rate in the ALPS group (20%) was 2.5 times lower than in the PHILOS group (48%). These findings corroborated early results reporting a complication rate of 22.6% on 31 patients treated with the ALPS plating system [40]. As it has been suggested that the incidence of avascular necrosis increased after 12 months [44], we reported late complications due to a follow-up period of 18 months or more (Fig. 4). This allows a more comprehensive understanding of short-term outcomes compared to previous studies which have shorter follow-up periods [39, 40]. Complication rate in the PHILOS group (48%) was comparable to some published studies (values up to 50% in 110 patients treated with the PHILOS plating system [45], and up to 49% in 514 patients [31]) but higher than some other locking plates (13% in 646 patients [46]).

The main complication rates (secondary displacement, avascular necrosis, screw perforation and subacromial impingement) are presented in Table 5. We found complication rates consistent with the literature, except for screw perforation in the PHILOS group. Whilst not statistically different, screw perforation rate was lower in the ALPS group, what may be explained due to the use of smooth blunt-ended pegs offered by the ALPS plating system instead of screws. Whilst not statistically different, subacromial impingement and stiffness rates were lower in the ALPS group, what may be explained due to the low profile offered by the ALPS plating system. Regarding infections, 0% were deep infections and 7% were low grade infections (the two cases were late infections caused by Propionibacterium acnes which was found after implant removal secondary to avascular necrosis).

**Table 5:** Comparison of main complication rates

	ALPS	PHILOS	Chen CY et al. [40]	Chen CY et al. [40]	Sproul RC et al. [31]
Number of patients	20	27	31	35	514
Plating system	ALPS	PHILOS	ALPS	PHILOS	various
Mean follow-up period (months)	21	21	13.3	13	29.2
Secondary displacement (varus malunion) (%)	10	19	6.5	17.1	16
Avascular necrosis (%)	10	7	3.2	17.1	10
Screw perforation (%)	10	22	6.5	8.6	7.5
Subacromial impingement (%)	0	11	0	11.4	6
Stiffness (%)	0	7	6.5	5.7	0.2

Implant removal secondary to avascular necrosis was the only cause requiring reoperation in the A group. On the other hand, causes for implant removal in the P group included avascular necrosis, screw perforation, impingement, or stiffness. In the literature, short term reoperation rates with the ALPS plating system were between 3.2% in 31 patients at 13 months [40] and 13% in 15 patients at 31.9 weeks [39]. This reoperation rate reached 18% with the PHILOS plating system at 2.5 years [45] and 14% in previous studies including several locking plates [31, 32]. Our results with the ALPS plating system were therefore consistent with recent studies and reported a lower reoperation rate than with other plates. Further studies with a bigger sample size are therefore required to confirm these rates.

To underline the strengths of this study, all surgeries were performed by the same fellowship trained upper extremity trauma surgeon in one single trauma center using identical surgical approach and rehabilitation protocol. All post-operative radiographic analyses were supervised by this same surgeon. The design of our study included a mean follow-up period of 21 months, which is longer than all previous studies involving the ALPS plating system and allowed us to report performance and complications on a longer period.

The first limitation is the retrospective nature of this study. Further randomized control trials are required to confirm the results presented here. The second limitation is the small sample size. Additionally, missing data in the reporting of ROM was converted to numerical value according to [42, 43] as described in paragraph II.C. Finally, although ROM was also collected before and after implant removal, this data was not compared between both groups due to the low number of patients who were affected in the ALPS group.

## Conclusion

With a total of 47 patients and a mean follow-up period of 21 months, this study allowed us to better estimate short-term outcomes and complications of the ALPS plating system. The ALPS plating system showed better clinical outcomes with faster recovery in abduction and external rotation, although no difference in ROM remained after 21 months. Additionally, the complications rate was lower at last follow up. In our experience, the ALPS plating system is an effective management option in some PHF.

## Data Availability

The datasets used during the current study are available from the corresponding author on reasonable request.

## Abbreviations

ALPS: Anatomic Locking Plate System
ES: Effect size
IQR: Interquartile range
OR: Odd ratio
PHF: Proximal humerus fracture
PHILOS: Proximal Humeral Internal Locking System
ROM: Ranges of motion
